# Supplementary material for: The Immunomodulatory Effects of Apigenin and Quercetin on Cytokine Secretion by the Human Gingival Fibroblast Cell Line and Their Potential Link to Alzheimer’s Disease
Source: Pharmaceuticals (Basel). 2025 Apr 26;18(5):628. doi: 10.3390/ph18050628 (PMC12114632; doi:10.3390/ph18050628)
Supplement: Supplementary file 1 [file pharmaceuticals-18-00628-s001.zip › pharmaceuticals-3529158-supplementary.pdf]

Supplementary informations

The Immunomodulatory Effects of Apigenin and Quercetin on Cytokine Secretion by the Human Gingival Fibroblast Cell Line and Their Potential Link to Alzheimer's Disease

Anna Kurek-Górecka [1,*], Małgorzata Kłósek [1], Radosław Balwierz [2], Grażyna Pietsz [1] and Zenon P. Czuba [1]

[1] Department of Microbiology and Immunology, Faculty of Medical Sciences, Medical University of Silesia in Katowice, Jordana 19, 41-808 Zabrze, Poland; mklosek@sum.edu.pl (M.K.); gpietsz@sum.edu.pl (G.P.); zczuba@sum.edu.pl (Z.P.C.)
[2] Institute of Chemistry, University of Opole, Oleska 48, 45-052 Opole, Poland; radoslaw.balwierz@uni.opole.pl
* Correspondence: akurekgorecka@sum.edu.pl; Tel.: +48-322-722-554

Table S1. Mean values and standard deviations (SD) of cell viability [%] in HGF-1 cells exposed to apigenin or quercetin at various concentrations (10–100 μM), alone or in combination with immunostimulants (LPS, IFN-α, LPS+IFN-α).

Sample	Mean	SD	Sample	Mean	SD
Cont. DMSO	109,8727	6,471021	Cont. DMSO	94,76549	3,705834
Apigenin 10μM	96,42349	5,516164	Quercetin 10μM	91,71566	1,197474
Apigenin 25μM	87,45174	3,803441	Quercetin 25μM	71,87206	1,472696
Apigenin 50μM	73,42004	3,917339	Quercetin 50μM	62,41769	1,425364
Apigenin 100μM	60,64824	2,317129	Quercetin 100μM	57,06726	2,062999
Cont. IFNα	93,699	2,842032	Cont. IFNα	95,71006	2,681317
Apigenin 10μM +IFNα	77,33051	4,178633	Quercetin 10μM +IFNα	76,75808	4,846852
Apigenin 25μM +IFNα	61,0248	4,568022	Quercetin 25μM +IFNα	61,5157	3,068528
Apigenin 50μM +IFNα	52,13434	2,775503	Quercetin 50μM +IFNα	52,89599	4,566621

Apigenin 100μM +IFNα	45,22913	1,608515	Quercetin 100μM +IFNα	57,45904	2,719696
Cont. LPS	100	2,333869	Cont. LPS	97,28402	3,036113
Apigenin 10μM +LPS	89,3191	1,615106	Quercetin 10μM +LPS	81,84665	1,798873
Apigenin 25μM +LPS	79,21237	1,269058	Quercetin 25μM +LPS	70,1	2,881268
Apigenin 50μM +LPS	60,27972	1,965988	Quercetin 50μM +LPS	61,2581	1,479431
Apigenin 100μM +LPS	47,15495	2,484059	Quercetin 100μM +LPS	56,16631	2,237034
Cont. LPS +IFNα	97,32143	2,020322	Cont. LPS +IFNα	95,4149	3,077234
Apigenin 10μM +LPS+IFNα	96,62967	3,338405	Quercetin 10μM +LPS+IFNα	89,66879	4,090438
Apigenin 25μM +LPS+IFNα	80,04344	3,498222	Quercetin 25μM +LPS+IFNα	65,76497	4,407906
Apigenin 50μM +LPS+IFNα	64,95335	4,390507	Quercetin 50μM +LPS+IFNα	51,94484	2,459013
Apigenin 100μM +LPS+IFNα	52,83945	3,380752	Quercetin 100μM +LPS+IFNα	46,43446	3,062616

Table S2. Mean concentrations [pg/mL], standard deviations (SD), and relative standard deviations (RSD, %) of cytokines (IL-1β, IL-6, IL-8, IL-15, TNF-α) in HGF-1 cell culture supernatants under various experimental conditions corresponding to Figure 4.

Sample	IL-1β	SD	RSD	IL-6	SD	RSD	IL-8	SD	RSD	IL-15	SD	RSD	TNF-α	SD	RSD
Cont. DMSO	1,042667	0,07852	7,530656	130,1533	25,73749	19,77475	24,34	24,71787	101,5524	1228,843	19,18535	1,561253	3,498772	0,327212	9,352195
Api 25µg/mL	1,314667	0,07852	5,972589	73,64667	22,30792	30,29047	11,58667	2,626506	22,66835	1542,423	103,0626	6,681864	3,641579	0,214211	5,882353
Api 50µg/mL	1,738	0,775482	44,61921	32,83	4,076261	12,41627	8,913333	1,377909	15,45896	1582,057	243,488	15,3906	5,179123	2,479906	47,88275
Que 25µg/mL	2,078519	0,313052	15,06131	77,48	28,90005	37,30001	34,22333	10,78993	31,528	1096,533	178,8754	16,31281	7,432857	1,666974	22,42709
Que 50µg/mL	2,259259	0,313052	13,85641	45,90333	10,53477	22,94991	31,53667	5,342587	16,94087	790,6667	347,8834	43,99875	7,26	0	0
Cont. IFN-α	1,269333	0,07852	6,185896	273,0067	70,04291	25,65612	14,37667	4,342146	30,20273	1567,06	52,70969	3,363604	3,784386	0,123675	3,26802
Api 25µg/mL +IFN-α	1,201333	0,03926	3,26802	164,49	15,10967	9,185768	81,04	37,99006	46,87815	1465,06	117,3666	8,011047	4,679298	2,941816	62,86874
Api 50µg/mL +IFN-α	1,178667	0,07852	6,661734	42,97667	5,090877	11,84568	19,10667	3,589517	18,78673	1377,017	46,60993	3,384849	3,570175	0,123675	3,464102
Que 25µg/mL + IFN-α	2,168889	0	0	473,7867	18,33883	3,870693	724,75	70,94858	9,789387	646,9133	144,1391	22,28106	5,877143	0,598795	10,18853
Que 50µg/mL + IFN-α	2,168889	0	0	152,2967	5,065001	3,325746	257,0567	33,30215	12,95518	1128,77	86,76549	7,686729	8,47	1,079493	12,74489
Cont. LPS	1,110667	0,03926	3,534798	171,97	78,87913	45,86796	23,47333	9,523468	40,57143	1534,39	32,6838	2,130084	6,865439	6,358493	92,61598
Api 25µg/mL + LPS	1,178667	0,07852	6,661734	136,55	6,341916	4,644391	79,38	26,50524	33,39032	1589,913	193,0324	12,14107	6,645263	2,415755	36,35304
Api 50µg/mL + LPS	1,539111	0,433377	28,15762	85,73333	96,01032	111,9872	23,58667	29,2472	123,9989	1220,427	91,59758	7,505374	4,573409	1,464157	32,01456
Que 25µg/mL + LPS	3,965926	3,124344	78,77969	69,11333	11,58617	16,76402	26,95333	9,176984	34,04768	922,87	355,7742	38,55085	7,432857	2,095781	28,19618
Que 50µg/mL + LPS	3,920741	3,158243	80,55221	47,74	4,263004	8,929626	29,93	2,04382	6,828667	1020,653	131,4717	12,88113	8,297143	1,037143	12,5
Cont. LPS + IFN-α	1,269333	0,07852	6,185896	38,17	1,643807	4,306541	10,85667	3,176261	29,25632	1612,043	107,8777	6,691986	3,498772	0,445915	12,74489
Api 25µg/mL + LPS+ IFN-α	1,088	0	0	20,96	5,718505	27,28294	6,236667	1,519616	24,36584	1622,39	55,13344	3,398285	6,044912	4,533719	75,00057
Api 50µg/mL + LPS+ IFN-α	1,224	0,136	11,11111	32,01333	4,982473	15,56374	8,546667	2,683903	31,40292	1601,843	137,3269	8,573054	5,32193	2,356353	44,27629
Que 25µg/mL + LPS+ IFN-α	1,807407	0,156526	8,660254	492,2167	49,21535	9,998717	582,1367	91,29531	15,6828	845,6633	330,3299	39,06164	6,395714	1,305043	20,40496
Que 50µg/mL + LPS + IFN-α	2,168889	0	0	219,1767	41,23055	18,81156	269,1533	156,3859	58,1029	589,1967	274,9614	46,66717	7,087143	1,305043	18,41423
